# Different types of interaction between PCNA and PIP boxes contribute to distinct cellular functions of Y-family DNA polymerases

**DOI:** 10.1093/nar/gkv712

**Published:** 2015-07-13

**Authors:** Yuji Masuda, Rie Kanao, Kentaro Kaji, Haruo Ohmori, Fumio Hanaoka, Chikahide Masutani

**Affiliations:** 1 Department of Genome Dynamics, Research Institute of Environmental Medicine, Nagoya University, Furo-cho, Chikusa-ku, Nagoya 464-8601, Japan; 2 Department of Toxicogenomics, Nagoya University Graduate School of Medicine, 65 Tsurumai-cho, Showa-ku, Nagoya 466-8550, Japan; 3 Department of Gene Information, Institute for Virus Research, Kyoto University, Sakyo-ku, Kyoto 606-8517, Japan; 4 Department of Life Science, Graduate School of Science, Gakushuin University, 1-5-1 Mejiro, Toshima-ku, Tokyo 171-8588, Japan

## Abstract

Translesion DNA synthesis (TLS) by the Y-family DNA polymerases Polη, Polι and Polκ, mediated via interaction with proliferating cell nuclear antigen (PCNA), is a crucial pathway that protects human cells against DNA damage. We report that Polη has three PCNA-interacting protein (PIP) boxes (PIP1, 2, 3) that contribute differentially to two distinct functions, stimulation of DNA synthesis and promotion of PCNA ubiquitination. The latter function is strongly associated with formation of nuclear Polη foci, which co-localize with PCNA. We also show that Polκ has two functionally distinct PIP boxes, like Polη, whereas Polι has a single PIP box involved in stimulation of DNA synthesis. All three polymerases were additionally stimulated by mono-ubiquitinated PCNA *in vitro*. The three PIP boxes and a ubiquitin-binding zinc-finger of Polη exert redundant and additive effects *in vivo* via distinct molecular mechanisms. These findings provide an integrated picture of the orchestration of TLS polymerases.

## INTRODUCTION

Translesion DNA synthesis (TLS), a DNA damage tolerance mechanism, is a crucial biological function that protects cells from various genotoxic agents. Particularly in humans, DNA polymerase η (Polη), a Y-family DNA polymerases (1), plays an important role in preventing cell death and mutagenesis after ultraviolet (UV) light irradiation, and malfunction of Polη causes the inherited genetic disorder, xeroderma pigmentosum variant (XP-V) (2–4).

Interactions between proliferating cell nuclear antigen (PCNA) and the three Y-family human DNA polymerases (Polη, Polι and Polκ) are critically involved in regulation of TLS. Polη and Polκ are known to contain two PCNA-interacting protein (PIP) boxes (PIP1 and PIP2) in their central and C-terminal regions, respectively, whereas Polι is known to contain only one functional PIP box (PIP1, in the central region) (5–12). In DNA-damaged cells, PCNA is mono-ubiquitinated at residue K164 by the RAD6–RAD18 complex (13–15), and poly-ubiquitinated by additional factors including UBC13, MMS2, and RAD5/HLTF or SHPRH (13,16–19). Each of the three Y-family DNA polymerases described above has one or two copies of the ubiquitin-binding domain (UBD), called UBZ (ubiquitin-binding zinc-finger) in Polη and Polκ and UBM (ubiquitin-binding motif) in Polι (6). These findings support the notion that mono-ubiquitination of PCNA plays a key role in switching from replicative DNA polymerase stalled at a site of DNA damage to a DNA polymerase (such as Polη, ι or κ) capable of carrying out TLS (14–15,20–21). However, this idea is still controversial (5), and more recent publications report that Polη and Polκ are able to carry out TLS independently of PCNA ubiquitination in some circumstances (22–24).

The intracellular functions of the various motifs of Polη are monitored in two ways: formation of nuclear foci containing Polη co-localized with PCNA and complementation of UV sensitivity of XP-V cells. Mutations in PIP2 strongly impair the localization of Polη, indicating that PIP2 plays a crucial role in the accumulation of this protein in replication foci (23,25–27). However, *ubz* mutants also failed to accumulate in replication foci (6,26), and accumulation is barely detectable in a human cell line expressing the PCNA^K164R^ mutant instead of endogenous PCNA (28) and in a PCNA^K164R^ knock-in murine cell line (23), demonstrating that the PIP2–PCNA interaction itself is not sufficient for foci formation. Recently, Durando and colleagues reported an additional function of PIP2 of Polη, namely, that Polη promotes mono-ubiquitination of PCNA in a PIP2-dependent manner (29). It remains unclear how the two PIP2-mediated activities, co-localization of Polη with PCNA and promotion of PCNA mono-ubiquitination, are linked at the molecular level.

The *pip2* and *ubz* mutants of Polη exhibit pronounced defects in foci formation, but retain the capacity to complement UV sensitivity of XP-V cells; however, the levels of complementation activity vary amongst studies by different groups (5–6,25–26,30), including one report that showed no role for PIP2 in survival after UV irradiation (27). Even a *pip2 ubz* double mutant (25,26) and a mutant in which PIP2 and UBZ were deleted (30) still exhibited significant levels of complementation activity. By contrast, the *pip1 pip2* double mutant and a mutant lacking the entire PIP1, UBZ and PIP2 domains were severely defective in complementation (5,30).

The biochemical activities of the motifs of human Polη have been studied *in vitro*. In primer extension assays, both PIP1 and PIP2 contributed to some extent to the stimulation of DNA synthesis in the presence of PCNA (5,8,30). Using a reconstitution system to investigate switching between Polδ and Polη at DNA lesions, we demonstrated that the weak enhancement of the recruitment of Polη to the 3′-end of primer DNA by the UBZ domain depended on mono-ubiquitination of PCNA (21). These biochemical properties of mutant proteins defective for PIP1, PIP2 or UBZ are well correlated with their abilities to complement UV sensitivity of XP-V cells, but not with their abilities to promote accumulation into the replication foci.

Here, we describe the molecular functions of Polη's PIP1, PIP2 and UBZ domains, together with the newly found PIP3, in UV tolerance, and present findings that resolve the controversies raised in previous reports. Our key finding is that Polη has two functionally distinct types of PIP box, one that stimulates DNA synthesis, and another that promotes PCNA mono-ubiquitination and accumulation into replication foci. Both PIP functions, together with the UBZ domain, are redundantly required for survival after UV irradiation. Additionally, we show that Polκ has two PIP boxes with different functions, whereas Polι has only one PIP box involved in stimulation of DNA synthesis. Taking the results of previous reports together with the *in vivo* and *in vitro* data obtained in this study, we propose a model for the cellular functions of the various motifs of Polη/ι/κ in orchestrating TLS.

## MATERIALS AND METHODS

### Proteins

Expression plasmids were constructed as follows. Human *POLH*, *POLI* (encoding 740 amino-acid residues) (11) and *POLK* were cloned into pET20b(+) (Novagen) to obtain untagged proteins, and into pET15b (Novagen) to obtain N-terminally histidine-tagged proteins. Plasmids for expression of Polη, Polη^ubz^ and His-Polη in *E. coli* were described previously (21,31). A truncated gene encoding PolηΔC (32) was cloned into pET21a(+) (Novagen). A gene encoding UBCH5c^S22R^ was cloned into pET15b. Mutations were created by PCR, and nucleotide sequences were verified after cloning.

E1, RAD6-(His-RAD18)_2_, RAD6-(RAD18^ΔC2^)_2_, ubiquitin, RPA, PCNA, RFC, His-Polη, Polη and their mutants were purified as described previously (21,31,33–35). Column chromatography was carried out at 4°C on an FPLC system (GE Healthcare Life Science) using columns from GE Healthcare unless otherwise indicated. Protein concentrations were determined by the Bio-Rad protein assay using BSA (Bio-Rad) as the standard.

PolηΔC was purified in the same way as Polη (21), except that HiTrap Phenyl HP was used instead of an Econopack methyl column (Bio-Rad).

A histidine-tagged Polκ (His-Polκ) and its mutants were purified as follows. BL21 (DE3) harbouring each of the expression plasmids and pMS-tRNA1 (36) was grown in 2 l of Terrific broth (37) supplemented with ampicillin (250 μg/ml) and kanamycin (30 μg/ml) at 15°C. His-Polκ was induced with 0.2 mM isopropyl β-_D_-thiogalactopyranoside (IPTG) for 8 h, and then purified by sequential chromatography on Ni^2+^-charged HiTrap chelating HP, POROS 50 HE (Applied Biosystems), Econopack CHT-II (BIO-RAD) and Superdex 200 columns. The peak fraction containing His-Polκ was frozen in liquid nitrogen and stored at −80°C.

Polι and Polι^pip1^ were purified as follows. BL21 (DE3) harbouring each of the expression plasmids was grown in 5 l of LB supplemented with ampicillin (250 μg/ml) at 15°C. Polι was induced with 0.2 mM IPTG for 5 h, and then purified by sequential chromatography on HiTrap Capto MMC, Ni^2+^-charged HiTrap chelating HP, HiTrap SP HP, HiTrap Q HP and Superdex 200 columns. Note that Polι itself (without the His-tag) has weak affinity for the Ni^2+^-charged HiTrap chelating column. For Polι^pip1^, the gel-filtration chromatography step was omitted. The peak fraction containing Polι was frozen in liquid nitrogen and stored at −80°C.

A histidine-tagged Polι (His-Polι) was purified as follows. BL21 (DE3) harbouring the expression plasmid was grown in 3 l of LB supplemented with ampicillin (250 μg/ml) at 15°C. Polι was induced with 0.2 mM IPTG for 8 h, and then purified by sequential chromatography on Ni^2+^-charged HiTrap chelating HP, HiTrap SP HP, HiTrap Q HP and Superdex 200 columns. The peak fraction containing His-Polι was frozen in liquid nitrogen and stored at −80°C.

His-UBCH5c^S22R^ was purified as follows. BL21 (DE3) harbouring the expression plasmid was grown in 2 l of LB supplemented with ampicillin (250 μg/ml) at 15°C. His-UBCH5c^S22R^ was induced with 0.2 mM IPTG for 16 h, and then purified by sequential chromatography on Ni^2+^-charged HiTrap chelating HP and HiTrap SP HP columns. The peak fraction containing His-UBCH5c^S22R^ was frozen in liquid nitrogen and stored at −80°C.

### PCNA-ubiquitination assays

PCNA-ubiquitination assays were performed as described (21). Briefly, the reaction mixture (25 μl) contained 20 mM HEPES-NaOH (pH 7.5), 50 mM NaCl, 0.2 mg/ml BSA, 1 mM DTT, 10 mM MgCl_2_, 1 mM ATP, poly(dA)-oligo(dT) (GE Healthcare) (100 ng), PCNA (1.0 pmol trimer), E1 (0.85 pmol), RAD6A-(His-RAD18)_2_ (0.54 pmol trimer), Ub (174 pmol) and DNA polymerases (2.5 pmol unless indicated otherwise). Reaction mixtures were prepared on ice, and then incubated at 30°C for 30 min unless indicated otherwise. The reactions were terminated with sample buffer for SDS-PAGE. Products were analysed by western blotting with anti-PCNA antibody (Santa Cruz Biotechnology, sc-7907). Signals were detected with a Chemi-Lumi One L kit (Nacalai Tesque, 07880–70) using ImageQuant™ LAS 4000 Mini Biomolecular Imager (GE Healthcare), and analysed using ImageQuant™ TL software (GE Healthcare).

### DNA polymerase assays

DNA polymerase assays were performed as described (35). Briefly, the reaction mixture (25 μl) contained 20 mM HEPES-NaOH (pH 7.5), 50 mM NaCl, 0.2 mg/ml BSA, 1 mM DTT, 10 mM MgCl_2_, 1 mM ATP, 0.1 mM of each deoxynucleotide (dGTP, dATP, dCTP and dTTP), 33 fmol of singly primed M13 mp18 ssDNA (the 5′-end ^32^P-labelled 36-mer primer, CAGGGTTTTCCCAGTCACGACGTTGTAAAACGACGG, is complementary to nt 6330–6295), RPA (9.1 pmol), RFC (260 fmol), PCNA (500 fmol trimer) and DNA polymerases (25 fmol for Polη and Polκ, 100 fmol for Polι, unless indicated otherwise). The proteins were combined on ice and incubated at 30°C for 10 min. The reactions were terminated with 2 μl of 300 mM EDTA, and the mixtures were immediately chilled on ice. After precipitation with ethanol, products were resolved on 10% polyacrylamide gels containing 7 M urea, and visualized using Typhoon FLA 9000 (GE Healthcare).

### Preparation of mono-ubiquitinated PCNA (mUb-PCNA)

PCNA was mono-ubiquitinated *in vitro* as described previously (38), with minor modifications (28). Briefly, a reaction mixture (800 μl) containing 50 mM Tris-base, 8 mM HEPES-NaOH (pH 7.5), 44 mM NaCl, 1 mM DTT, 10 mM MgCl_2_, 3 mM ATP, PCNA (13 nmol as trimer), E1 (110 pmol), His-UBCH5c^S22R^ (19 nmol) and ubiquitin (36 nmol) were incubated at 37°C for 15 min. Then, 19 nmol of ubiquitin was additionally introduced into the reaction mixture. After an additional 105 min incubation, mUb-PCNA was immediately purified by gel filtration on a Superdex 200 column. The peak fraction containing mUb-PCNA was frozen in liquid nitrogen and stored at −80°C.

### PCNA pull-down assays

Four microlitres of MagneticHis™ Ni Particles (Promega V8560) were re-suspended in 10 μl of a binding buffer containing 20 mM HEPES-NaOH (pH 7.5), 50 mM NaCl, 10 mM imidazole, 0.2 mg/ml BSA and 1 mM DTT, and then incubated at 4°C for 5 min with 10 pmol of each of the polymerases. After washing the beads twice with 50 μl of the binding buffer, 2.5 pmol of PCNA or mUb-PCNA was introduced and incubated at 4°C for 5 min in 25 μl of binding buffer. After the beads were washed twice with 50 μl of binding buffer, proteins that bound to the beads were analysed by western blotting with anti-PCNA antibody as described above.

### Cell lines and cultures

SV-40 immortalized XP-V fibroblasts (XP2SASV3) and a SV-40 immortalized normal human fibroblasts (WI38VA13) were maintained in Dulbecco's modified Eagle's medium (DMEM) supplemented with 10% fetal bovine serum (FBS), 0.584 g/L L-glutamine, 0.07 g/L penicillin and 0.15 g/L streptomycin. To obtain stably expressing cells, either wild-type or mutant Polη expression constructs were transfected into XP-V cells using the Neon® transfection system (Invitrogen), followed by 0.2 mg/ml G418 selection. For construction of expression plasmids in human cells, the indicated genes were cloned into pIRESneo2 (Clontech) to create N-terminally FLAG-tagged proteins or pAcGFP1-Hyg-C1 (Clontech) to create GFP fusion proteins, as described previously (28).

### Preparation of cellular fractions and western blotting

XP2SASV3 cells were transfected with expression constructs encoding either wild-type or mutant FLAG-Polη using the Neon® transfection system (Invitrogen) and incubated for 24 h. Three hours after 15 J/m^2^ UVC irradiation, cells were harvested and lysed in 1% SDS in PBS to obtain whole cell lysates (WCL). In the case of fractionation, cells were suspended in lysis buffer [20 mM Tris-HCl (pH 7.5), 150 mM KCl, 25% glycerol, 0.5% NP-40, 1.5 mM MgCl_2_, 1× Complete Protease Inhibitor Cocktail (Roche), 1× Phosphatase Inhibitor Cocktail Set II (Calbiochem)], and a portion was withdrawn as WCL. Next, soluble materials (soluble fractions) were separated by centrifugation. The precipitants were resuspended in micrococcal nuclease buffer [20 mM Tris-HCl (pH 7.5), 100 mM KCl, 300 mM sucrose, 0.1% Triton X-100, 2 mM MgCl_2_, 1 mM CaCl_2_, 1× EDTA-free Complete protease inhibitor cocktail (Roche)] and incubated with 2.5 U of micrococcal nuclease (Roche) at room temperature for 10 min. After centrifugation, soluble materials were collected as chromatin fractions, and precipitates (insoluble fractions) were resuspended in 1% SDS in PBS and solubilized by sonication. Cellular fractions were analysed by western blotting with anti-PCNA (Santa Cruz Biotechnology, sc-7907 or sc-56), anti-Polη (39), anti-Lamin B (Santa Cruz Biotechnology, sc-6216), anti-FLAG (M2 SIGMA, F1804) or anti-GFP (MBL, M048–3) antibodies.

### Analysis of co-localization of Polη with PCNA

XP2SASV3 cells were transfected with expression constructs encoding either wild-type or mutant FLAG-Polη using the Neon® transfection system (Invitrogen). Forty-eight hours after transfection, cells were irradiated with 15 J/m^2^ UVC and incubated for 3 h. Triton-soluble materials were removed by incubation with extraction buffer (0.5% Triton X-100, PBS, 0.4 μg/ml antipain, 0.4 μg/ml aprotinin, 0.2 μg/ml leupeptin, 0.16 μg/ml pepstatin, 0.1 mM EGTA and 0.5 mM phenylmethylsulfonyl fluoride), and then the cells were fixed with 3.5% formaldehyde in PBS and permeabilized with 0.1% Triton X-100 and 3.5% formaldehyde in PBS. After sequential treatments with 70% EtOH, 100% EtOH, and acetone on ice, cells were incubated with anti-POLH (Santa Cruz Biotechnology, sc-5592) and anti-PCNA (Santa Cruz Biotechnology, sc-56) antibodies. Alexa Fluor 488- and 594-conjugated secondary antibodies (Invitrogen) were used to visualize the immune-conjugated proteins. Nuclei were visualized by staining with 2 μg/ml Hoechst 33342. Images were collected using an LSM710 confocal microscope (Zeiss).

## RESULTS

### Promotion of PCNA mono-ubiquitination by Polη is dependent on PIP2 and PIP3, but independent of PIP1

**Figure 1.**
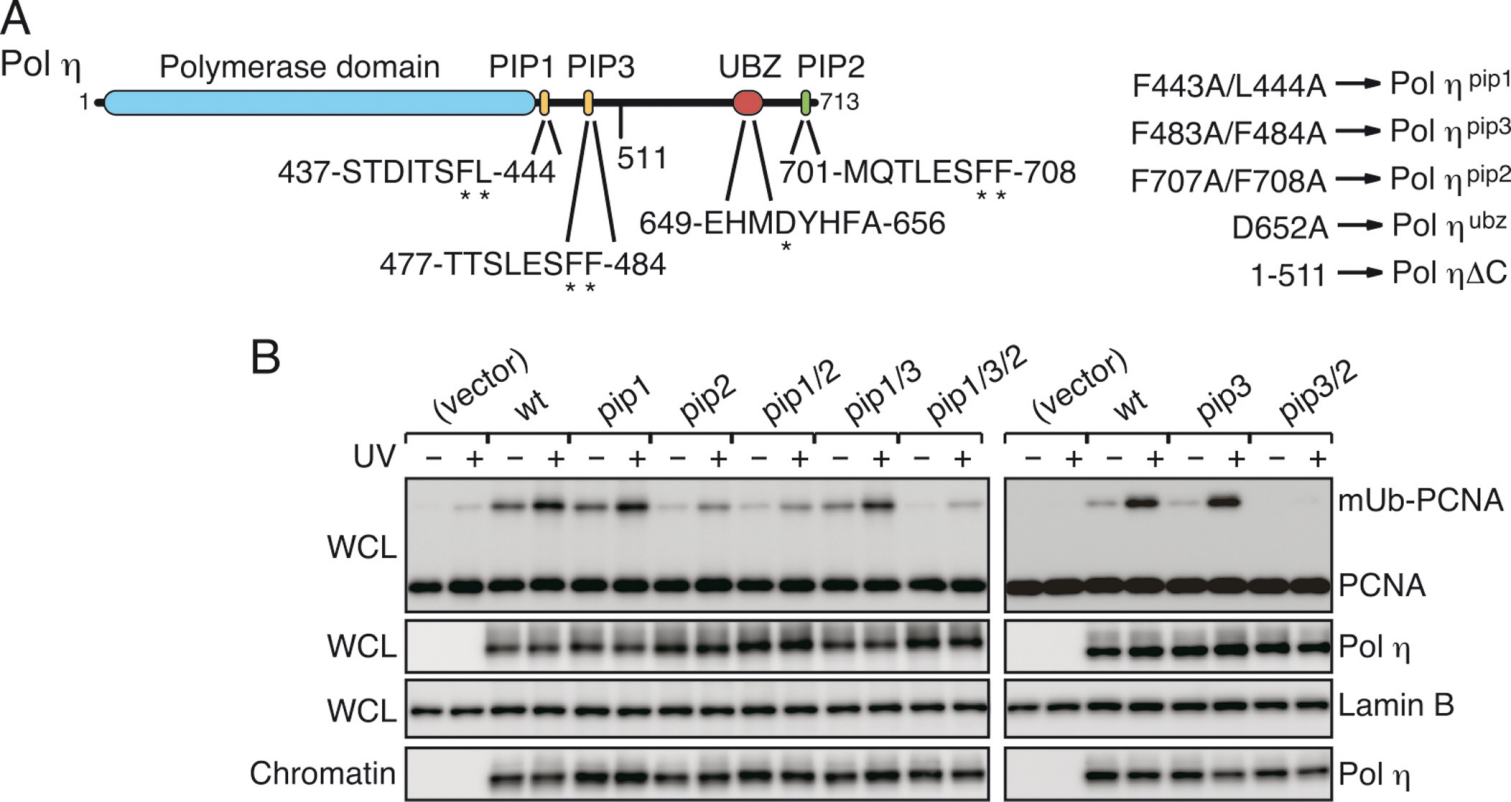
Polη promotion of PCNA ubiquitination depends on PIP3 and PIP2, but not on PIP1. **(A)** Schematic structure of human Polη. Parts of PIP and UBZ sequences are shown. Amino-acid residues indicated by asterisks were replaced with alanines in the mutants. **(B)** Western blot analysis of FLAG-Polη-expressing cells. XP-V cells were transfected with the indicated plasmids for expression of FLAG-Polη (wt) or the indicated *pip* mutants, incubated for 24 h, irradiated with UV (15 J/m^2^) and further incubated for 3 h. Whole-cell lysates (WCL) or chromatin fractions were subjected to western blotting with anti-PCNA, anti-Polη and anti-Lamin B (loading control) antibodies.

The three human Y-family DNA polymerases (Polη, ι and κ) share a basic architecture. Each protein contains a catalytic domain in the N-terminal half and various motifs/domains involved in interactions with other proteins in the C-terminal half (see Figures 1A and 4A, D). Human Polη contains multiple PIP boxes and a single copy of UBZ, which are believed to be involved in the interaction with mUb-PCNA in DNA-damaged cells. Recently, Durando and co-workers reported that depletion of endogenous Polη decreases the levels of damage-induced mUb-PCNA, and ectopically expressed Polη promotes mono-ubiquitination of PCNA in cells in a manner that depends on PIP2 at the C-terminus (29). However, those authors did not examine the contribution of another PIP box, PIP1, which is located in an internal region (5) (Figure 1A). To determine whether PIP1 plays any role in the promotion of PCNA mono-ubiquitination, we introduced the *pip1* or *pip2* mutation into FLAG-tagged Polη (Figure 1A), expressed the mutant proteins in XP-V cells, and analysed the levels of mUb-PCNA in cells by western blotting. As shown in Figure 1B, the result indicated that the *pip1* mutant and the wild type promoted mono-ubiquitination of PCNA to similar extents. Importantly, PCNA ubiquitination was observed in the presence or absence of UV irradiation, although it was more extensive when the cells were UV-irradiated. By contrast, the *pip2* mutant lost most of the ability to promote mono-ubiquitination. Because the *pip1 pip2* double mutant still exhibited weak activity, similar to that of the single *pip2* mutant, the residual activity could be attributable to additional PIP box(es). To identify another PIP box in Polη, we employed yeast two-hybrid assays and found one additional PIP box (hereafter, referred to as PIP3), which overlaps with a REV1-interacting region (RIR) (11,40) (Figure 1A; Supplementary Figure S1). Although short peptides carrying the PIP2 sequence interact with PCNA strongly enough for detailed physicochemical and structural analyses (10), the PCNA-binding activity of short peptides carrying the PIP1 sequence has never been detected, even using very sensitive yeast two-hybrid assay (11). Similarly, the PCNA-binding activity of PIP3 is not detected using short fragments; however, the activity of PIP3 appears stronger than that of PIP1, because the *pip3* mutation caused a much more drastic reduction in the positive signal in the yeast two-hybrid assay than the *pip1* mutation (Supplementary Figure S1). Subsequently, we made a series of *pip3* mutants and expressed them in XP-V cells. As expected, the residual activities of the *pip2* single and *pip1 pip2* double mutants were diminished further by the additional introduction of the *pip3* mutation. The levels of mUb-PCNA in *pip3 pip2* double and *pip1 pip3 pip2* triple mutants were similar to that in the vector control. On the other hand, the levels of mUb-PCNA in *pip3* single and *pip1 pip3* double mutants exhibited marginal differences from those in the wild type only in UV-unirradiated samples. Because all of these mutants were similarly detected in the chromatin fraction, the defects in the mutants were not attributed to alteration in sub-cellular localization (Figure 1B, bottom panel). These results indicate that PIP2 and PIP3 play a major and minor role, respectively, whereas PIP1 has no or little role, in promoting mono-ubiquitination of PCNA in cells, suggesting that two functional types of PIP boxes play distinct roles in the regulation of Polη.

Additionally, we showed that the *ubz* mutants severely reduced the ability to promote PCNA mono-ubiquitination (Supplementary Figure S2). However, these mutants had a significantly reduced ability to accumulate in chromatin (26) (Supplementary Figure S2), indicating that the mutations have additional effects, as postulated previously (5). Because the defect in promoting PCNA mono-ubiquitination could be also attributed to additional effects, such as alteration of sub-cellular localization, it remains unclear whether UBZ has the potential to promote ubiquitination of PCNA *in vivo*.

### Reconstitution of Polη-dependent mono-ubiquitination of PCNA *in vitro*

**Figure 2.**
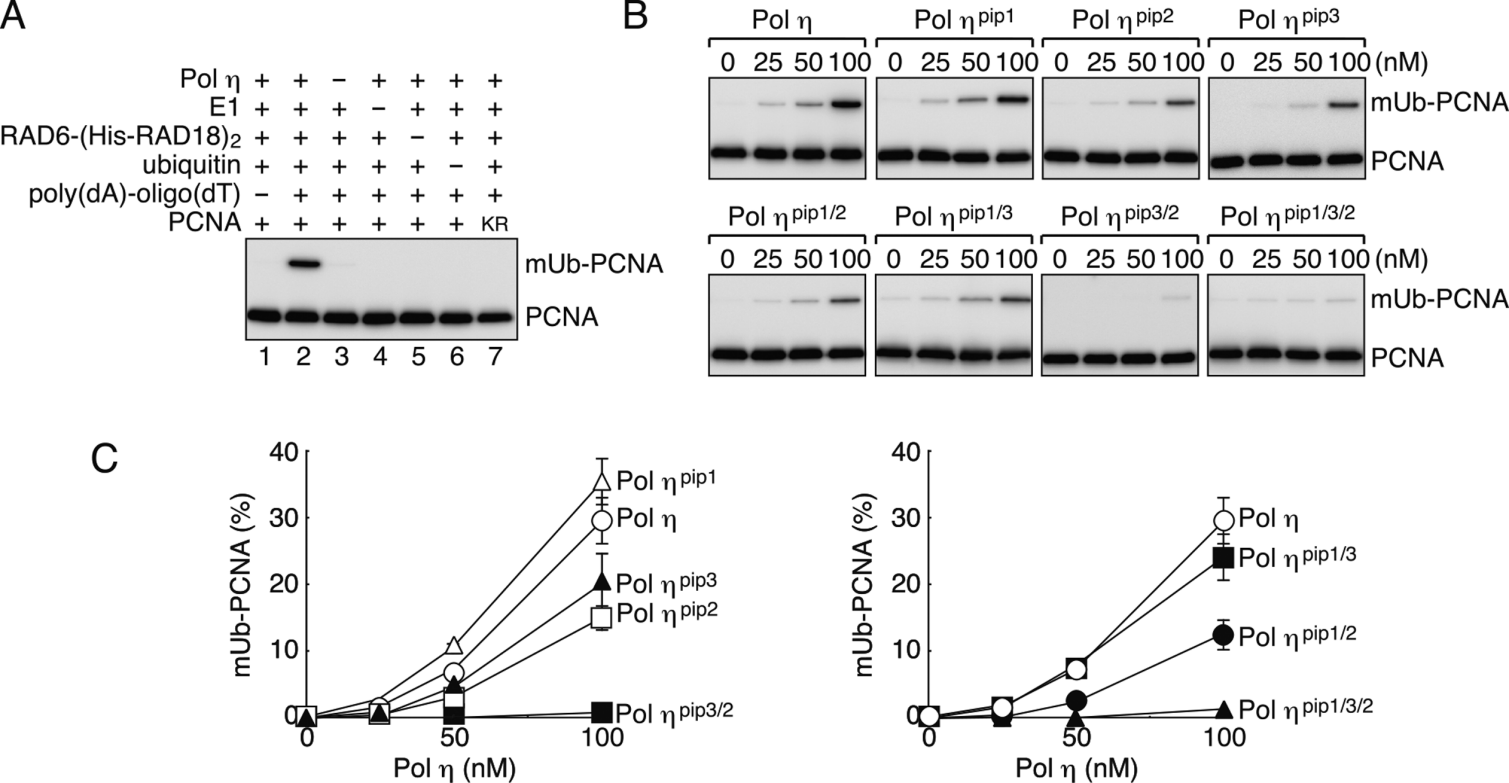
*In vitro* reconstitution of Polη–dependent PCNA ubiquitination. **(A)** Mono-ubiquitination reactions of PCNA were reconstituted with the indicated factors. Reaction products were analysed by western blotting with an anti-PCNA antibody. KR indicates the PCNA^K164R^ mutant. **(B)** Titration of Polη and its *pip* mutants. Indicated mutants were subjected to the ubiquitination assays as shown in (A). **(C)**Relative amounts of ubiquitinated PCNA were measured from gel images of more than three independent experiments, and the average values are plotted in the graph. Error bars show SD.

To study the molecular mechanisms underlying the promotion of PCNA mono-ubiquitination, we sought to develop *in vitro* experimental conditions for recapitulating the *in vivo* situation using purified enzymes (Supplementary Figure S3A) (21,34). Because Polη interacts with both RAD18 and PCNA (8,15,41), we first checked the possibility that such protein–protein interactions could themselves promote PCNA ubiquitination. However, we observed no ubiquitination of PCNA under such conditions (Figure 2A, lane 1). When poly(dA)-oligo(dT) was added into the reaction mixture, PCNA ubiquitination occurred and it was dependent on Polη (lanes 2 and 3) as well as on E1, RAD6-(His-RAD18)_2_ and ubiquitin (lanes 4–6). No modification was observed with the PCNA^K164R^ mutant, confirming that ubiquitination of PCNA takes place at Lys164 (lane 7). Note that PCNA is spontaneously loaded from the ends of poly(dA)-oligo(dT) without RFC in these experiments (21). Additionally, we found that the specific interaction between RAD18 and Polη was not required for promotion of PCNA mono-ubiquitination in the *in vitro* reactions, because a mutant of RAD18 lacking the C-terminal region required for its interaction with Polη (15,34) could promote PCNA ubiquitination as efficiently as full-length RAD18 (Supplementary Figure S4A). These results suggested that a certain mode of Polη–PCNA interaction on DNA is required for the promotion of PCNA ubiquitination, inspiring us to further investigate the mechanism.

### Effects of *pip* and *ubz* mutations on the promotion of *in vitro* PCNA ubiquitination

Given that Polη promotes PCNA ubiquitination *in vitro* through direct interaction with PCNA on DNA, we next asked whether one or all of the PIP boxes are required for promotion of ubiquitination. To address this question, the *pip* mutants of Polη used for the *in vivo* experiments described above (Figure 1; Supplementary Figure S3A) were examined for the ability to promote ubiquitination *in vitro*. The results demonstrated that *in vitro* promotion of ubiquitination is dependent on PIP3 and PIP2, but independent of PIP1 (Figure 2B, C), in good agreement with the *in vivo* observations (Figure 1B). Together, these results support the interpretation that the *in vivo* accumulation of mUb-PCNA is a consequence of directly promoting *de novo* ubiquitination by Polη.

In addition, we demonstrated that the *ubz* mutant promoted mono-ubiquitination as efficiently as the wild type (Supplementary Figure S4B), indicating that the UBZ function is dispensable for promotion of ubiquitination in our *in vitro* system.

### Different roles of the three PIP boxes of Polη in stimulation of DNA synthesis

**Figure 3.**
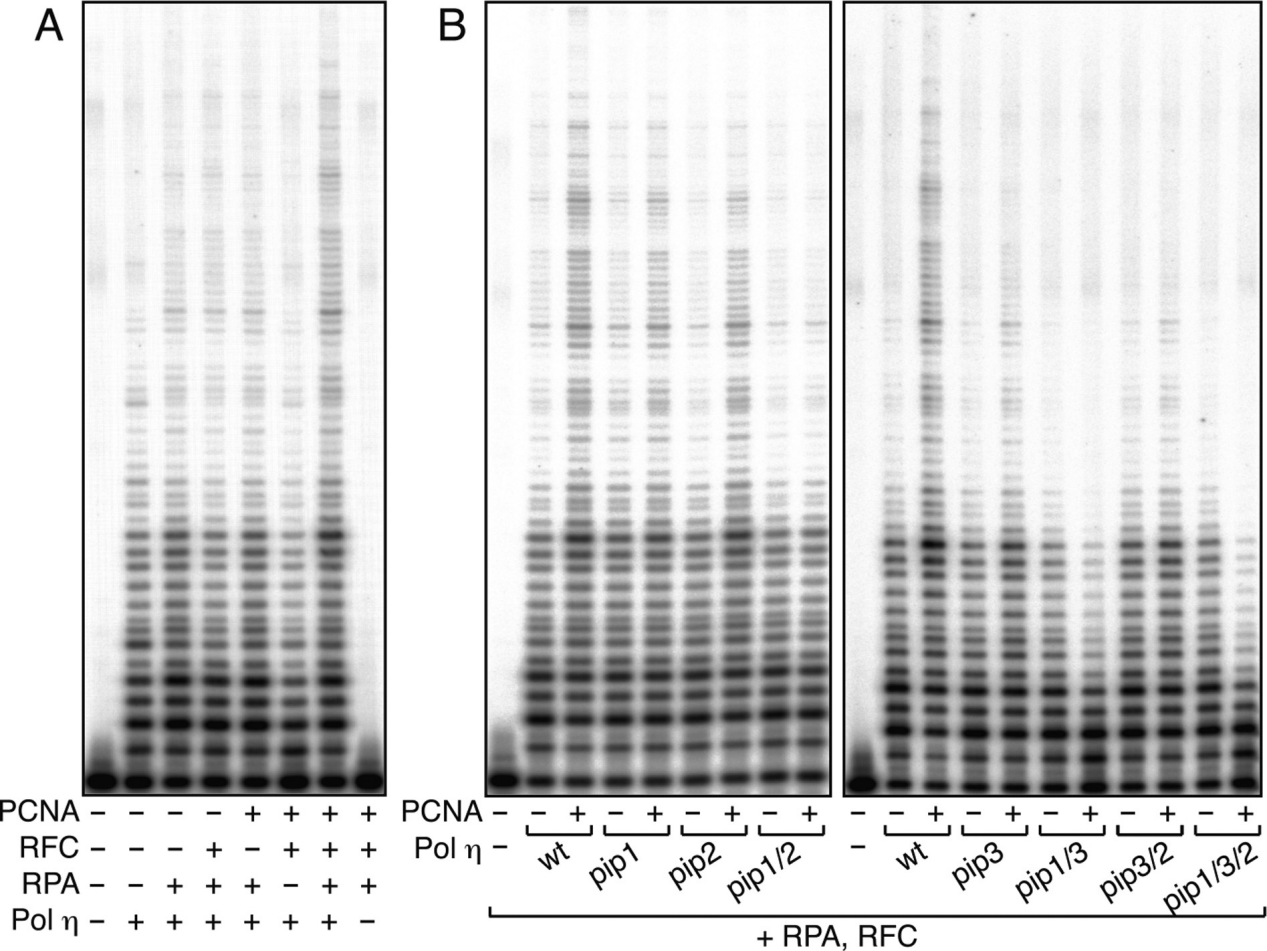
DNA polymerase assays of Polη in a reconstituted system *in vitro*. **(A)** DNA replication reactions using singly primed M13 mp18 ssDNA were reconstituted with the indicated factors. The reaction products were resolved in 10% polyacrylamide gels containing 7 M urea, and visualized using a PhosphorImager. **(B)** Analysis of *pip* mutants of Polη. Indicated mutants were subjected to replication assays shown in (A) in the presence or absence of PCNA.

Next, to study how PCNA stimulates the polymerase activity of Polη, we employed the primer extension assay using M13 mp18 ssDNA as a template in the presence of RPA and RFC. As shown in Figure 3A, we clearly detected stimulation of Polη polymerase activity by PCNA. When the *pip* mutants were examined, the stimulation was slightly reduced in each of the single *pip* mutants relative to the wild type (Figure 3B), indicating that all of the PIP boxes contribute to stimulation to some extent. The activities of the *pip1 pip2* and *pip3 pip2* were further reduced. Surprisingly, the activity of the *pip1 pip3* double mutant was inhibited by addition of PCNA, despite the fact that it still contains a PIP2 domain (Figure 3B). Similar inhibition was also observed with the *pip1 pip3 pip2* triple mutant (Figure 3B). Based on these results, we conclude that PCNA binding to PIP1 or PIP3, both located in the adjacent region of the Polη catalytic domain, is critical for the stimulation of DNA polymerase activity, and that even if PCNA binds to PIP2 at the C-terminus, PCNA does not stimulate Polη polymerase activity *in vitro* unless PIP1 or PIP3 is present. These results suggest that the function of PIP2 in stimulation of DNA synthesis is largely PIP1- and PIP3-dependent.

### Functional roles of PIP boxes of Polι and Polκ

**Figure 4.**
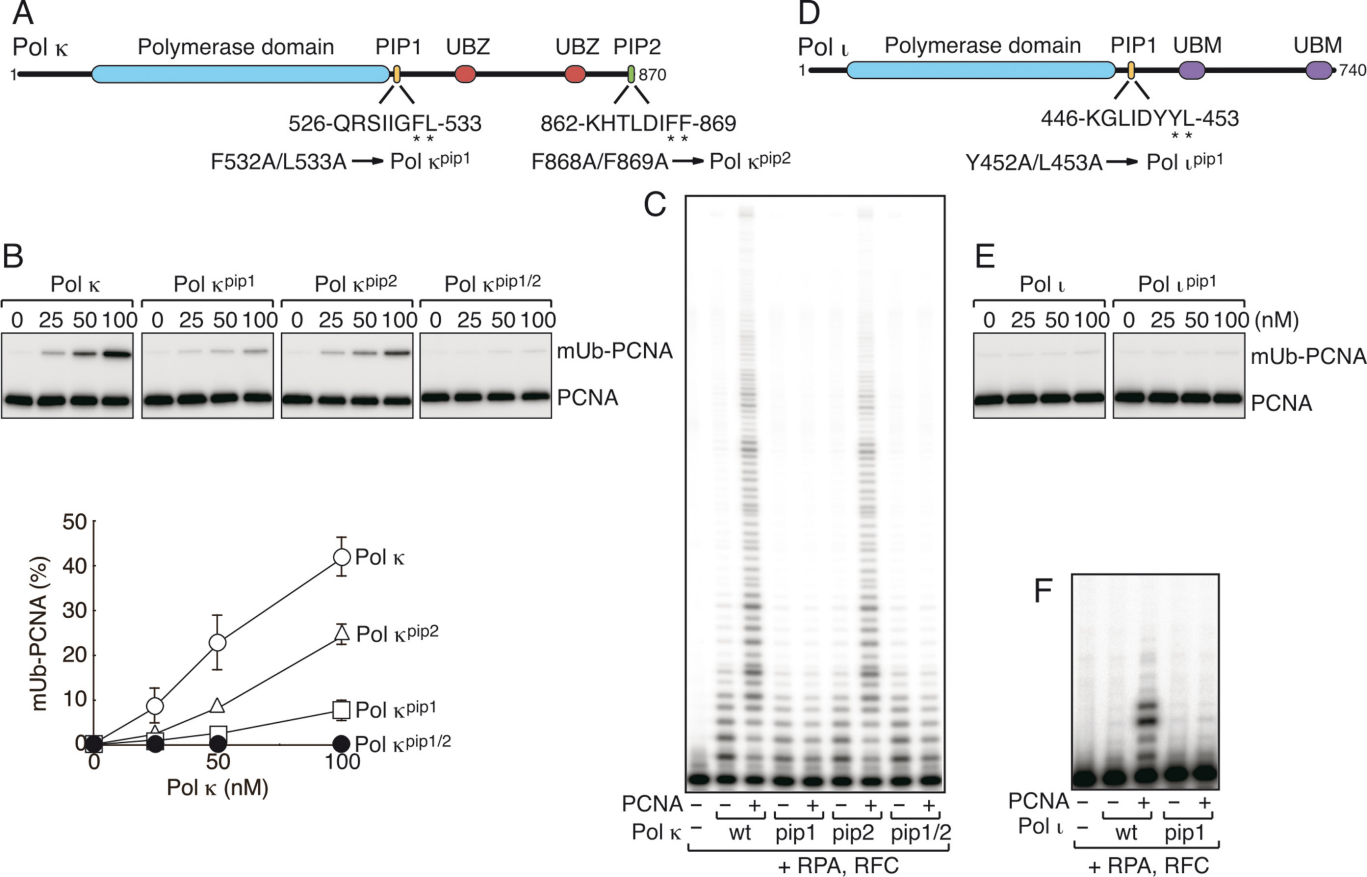
Analysis of Polκ, Polι and their *pip* mutants *in vitro*. **(A, D)** Schematic structures of human Polκ (A) and Polι (D), as shown in Figure 1A. **(B, E)** PCNA ubiquitination assays of His-Polκ (B) and Polι (E), as shown in Figure 2. **(C, F)** DNA polymerase assays of His-Polκ (C) and Polι (F), as shown in Figure 3.

The findings described above regarding the PIP boxes of Polη prompted us to investigate the PIP boxes of two other Y-family DNA polymerases, Polκ and Polι. Polκ has a PIP box at the C-terminus, which is required for formation of nuclear foci in cells with DNA damage (42), and has the potential to promote mono-ubiquitination of PCNA *in vivo* when Polκ is ectopically expressed (29). More recently, another PIP box was found adjacent to the catalytic domain (Figure 4A; Supplementary Figure S5); therefore, the internal PIP box was named PIP1, and the C-terminal one was renamed PIP2, following the example of Polη (Figure 4A) (11). Unlike the internal PIP1 and PIP3 boxes in Polη, the PCNA-binding activity of the internal PIP1 box in Polκ was detected in short fragments by the yeast two-hybrid assay, implying that the activity is equivalent to that of the C-terminal PIP2 in Polκ (Supplementary Figure S5). When the wild-type Polκ protein was introduced into the *in vitro* PCNA ubiquitination reaction, a large amount of ubiquitinated PCNA was observed (the leftmost panel in Figure 4B), as in the case of Polη. To study the roles of the respective PIP boxes, we examined the *pip1*, *pip2* and double mutants (Figure 4A; Supplementary Figure S3B). As shown in Figure 4B, the *pip1* and *pip2* mutants exhibited reduced levels of PCNA ubiquitination: the *pip2* mutant retained a relatively higher level, whereas the double mutant lost the activity. These results indicate that each of the two PIP boxes functions independently and have similar affinity for PCNA, and that both are required to promote the maximum level of ubiquitination (Figure 4B). Next, we examined effects of PCNA on DNA polymerase activity of the wild-type and mutant Polκ proteins. As shown in Figure 4C, the *pip2* mutant was stimulated as efficiently as the wild type by PCNA, but *pip1* and the double mutants failed to be stimulated by PCNA, indicating that the internal PIP1 box is responsible for stimulation by PCNA but the C-terminal PIP2 is not. Therefore, we conclude that the multiple PIP boxes of Polκ serve different functions: PIP1 stimulates DNA synthesis by PCNA, and PIP1 and PIP2 promote PCNA ubiquitination.

Next, we examined Polι (Figure 4D; Supplementary Figure S3C). In contrast to Polη and Polκ, Polι did not promote PCNA ubiquitination *in vitro* (Figure 4E). On the other hand, PCNA stimulated DNA synthesis of Polι *in vitro* in a PIP1-dependent manner (Figure 4F), in line with previous reports that Polι has only one functional PIP box (for stimulation of DNA synthesis) immediately adjacent to the catalytic domain (7,10,12).

**Figure 5.**
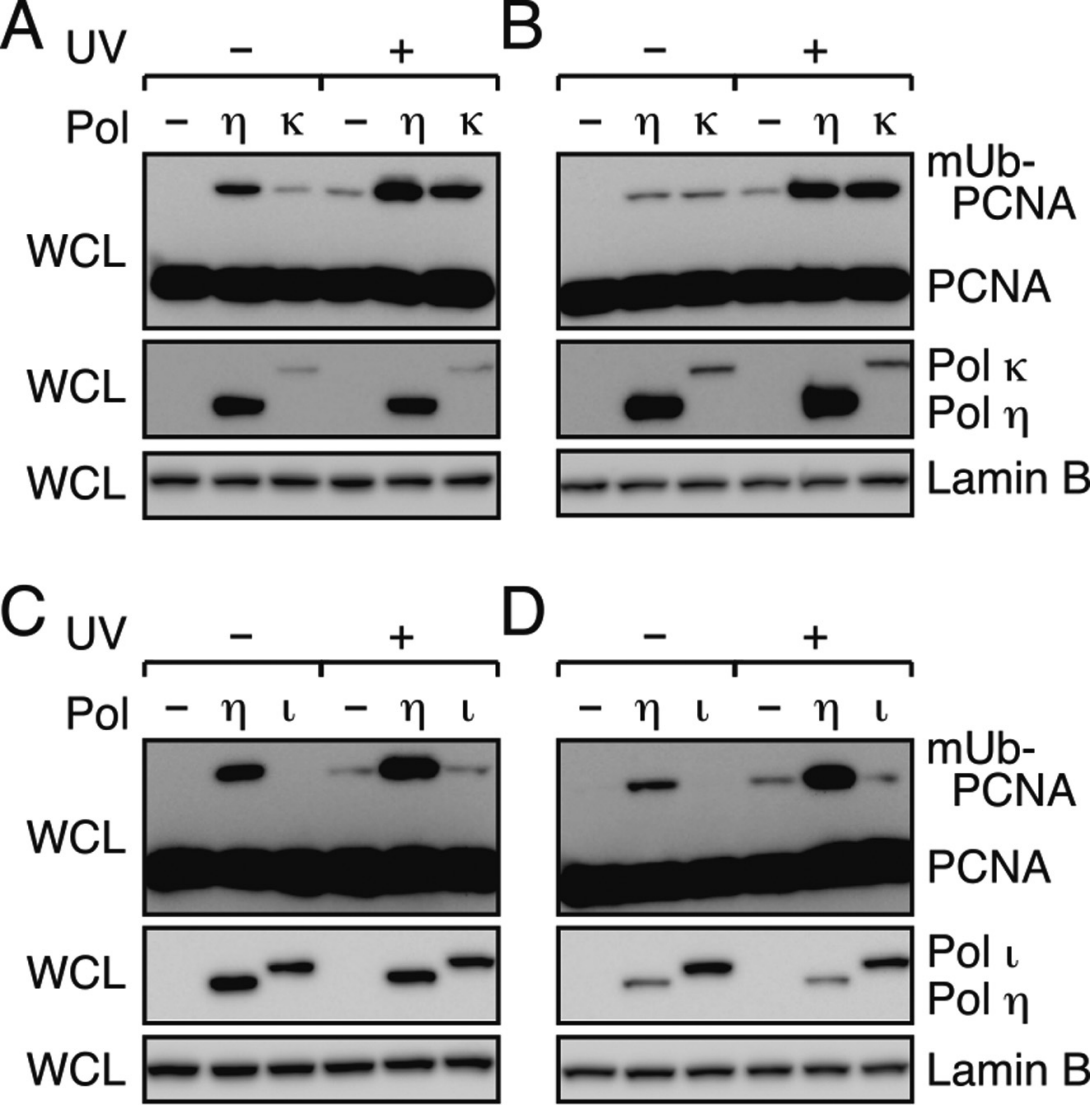
Promotion of mono-ubiquitination of PCNA in cells by Polκ but not Polι. **(A, B)** Western blot analysis of Polκ-expressing cells. XP-V (A) and normal cells (B) were transfected with a plasmid to express GFP-Polκ or GFP-Polη (as a control). **(C, D)** Western blot analysis of Polι-expressing cells. XP-V (C) and normal cells (D) were transfected with a plasmid to express FLAG-Polι or FLAG-Polη (as a control). The transfected cells were incubated for 24 h, irradiated with UV (15 J/m^2^) and further incubated for 3 h. Whole-cell lysates (WCL) were subjected to western blotting with anti-PCNA, anti-Lamin B (loading control) and anti-GFP or anti-FLAG antibodies.

Subsequently, we examined the levels of mUb-PCNA in cells with ectopic expression of Polκ or Polι, with or without UV irradiation (Figure 5). When GFP-Polκ was expressed in Polη-deficient (XP-V) or proficient cells, promotion of PCNA mono-ubiquitination was observed in both types of cells (Figure 5A, B). The promoting effect of ectopic expression of Polκ is weaker than that of Polη reported previously (29,43). The difference in the extent to which PCNA mono-ubiquitination was promoted by these enzymes could be attributed to differences in the expression system and/or cell types used in these experiments. Indeed, a difference is evident between the two types of cells used in our study (Figure 5A, B), indicating that it may not be appropriate to compare and draw conclusions from differences in mUb-PCNA levels between different cell lines. By contrast to Polη and Polκ, ectopic expression of FLAG-Polι in both cells did not increase the levels of mUb-PCNA (Figure 5C, D). These results are consistent with the *in vitro* properties described above (Figure 4B, E), implying that Polκ, but not Polι, can promote *de novo* mono-ubiquitination of PCNA *in vivo*.

### Interactions of Polη, ι and κ with mUb-PCNA

**Figure 6.**
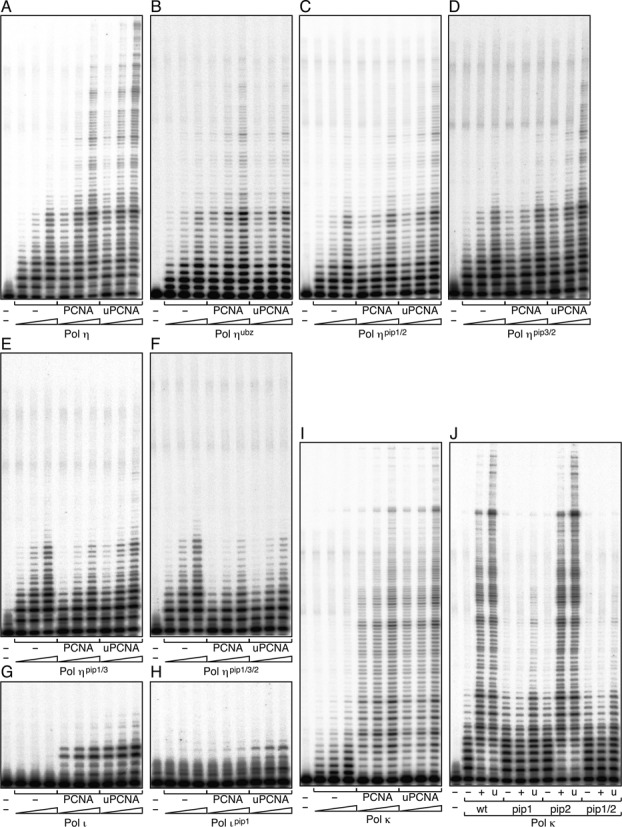
Interactions between Y-family DNA polymerases and mUb-PCNA *in vitro*. **(A–J)** Analysis of DNA synthesis by Polη (A–F), Polι (G–H) and His-Polκ (I–J), as shown in Figure 3, in the absence or presence of PCNA (designated as PCNA or +) or mUb-PCNA (designated as uPCNA or u). Concentrations of polymerases increase in the order 0.25, 0.5, and 1 nM (A–F and I) or 1, 2, and 4 nM (G–H), or remain constant at 1 nM (J).

PCNA is mono-ubiquitinated in DNA-damaged cells. Consequently, Y-family DNA polymerases with UBD(s), as well as PIP box(es), could interact with modified PCNA in preference to unmodified PCNA. To test this *in vitro*, we compared the stimulatory effects of mUb-PCNA and unmodified PCNA on DNA synthesis *in vitro*, using a primer extension assay (Figure 6; Supplementary Figure S3D). As shown in Figure 6A, mUb-PCNA stimulated DNA synthesis by Polη more effectively than unmodified PCNA under our assay conditions. As expected, no additional stimulation was observed with the *ubz* mutant of Polη (Figure 6B). DNA synthesis by either the *pip1 pip2* or *pip3 pip2* double mutants was stimulated by mUb-PCNA, although it was only marginally stimulated by unmodified PCNA (Figure 6C, D). The activity of the *pip1 pip3* double mutant was slightly higher in the presence of mUb-PCNA than in the absence of PCNA (Figure 6E). The *pip1 pip3 pip2* triple mutant exhibited a negative effect by the addition of mUb-PCNA (Figure 6F). These results suggested that UBZ function requires at least one PIP.

Next, we examined the stimulation of DNA synthesis by Polι and Polκ by mUb-PCNA. Additional stimulation, albeit marginal, by mUb-PCNA was reproducibly observed for Polι (Figure 6G) as well as its *pip1* mutant (Figure 6H). Similarly, mUb-PCNA stimulated Polκ and its *pip* mutants to a slightly greater extent than unmodified PCNA (Figure 6I, J). The relatively lower contributions of ubiquitin moieties to Polι and Polκ activity than to Polη activity could be attributed to the weaker affinity of Polι and Polκ for mUb-PCNA than Polη (Supplementary Figure S6).

### Cellular functions of the motifs of Polη

**Figure 7.**
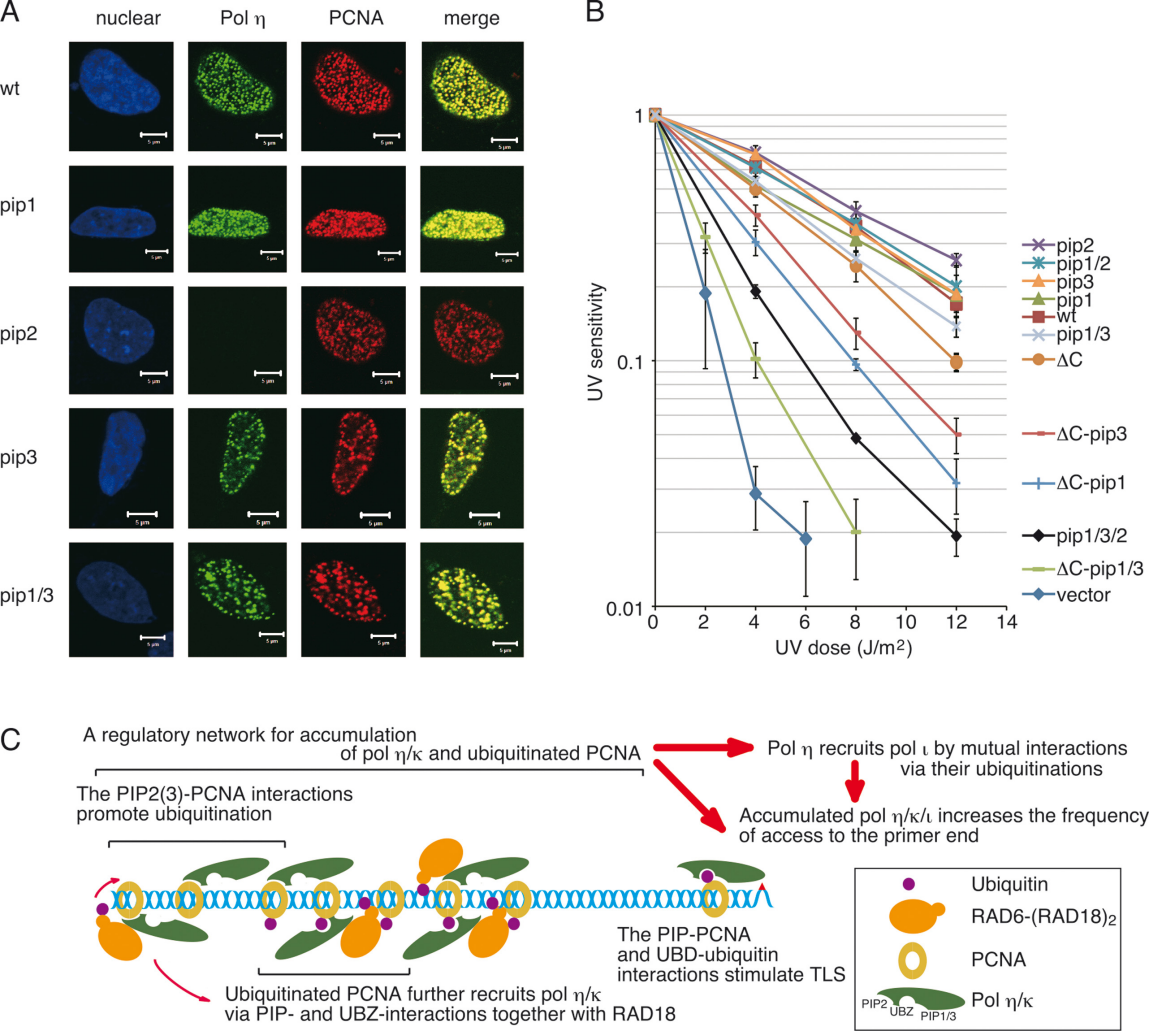
Cellular functions of the motifs of Polη. **(A)** Co-localization of Polη with PCNA. XP-V cells were transiently transfected with plasmids encoding wild-type FLAG-Polη or the indicated mutants. After UV irradiation, FLAG-Polη and PCNA were visualized by immunostaining with anti-Polη and anti-PCNA antibodies, respectively. Nuclei were stained by Hoechst 33342. Scale bars represent 5 μm. Control experiments confirming expressions of FLAG-Polη were shown in Supplementary Figure S7. **(B)** UV sensitivities of XP-V cells stably expressing FLAG-Polη. Cells were irradiated with the indicated dose of UVC, incubated with 1 mM caffeine for 4 days, and their viabilities were measured. Error bars show SD from three independent experiments. **(C)** A model for a regulatory network for foci formation and the TLS function of Polη/ι/κ. Interactions of Polη/κ with PCNA, together with RAD6-(RAD18)_2_, leads to their accumulation by promoting mono-ubiquitination of PCNA around stalled 3′-OH ends. Interactions of Polη/ι/κ with mUb-PCNA via PIPs and UBDs stimulate DNA synthesis at stalled 3′-OH ends. See text for details.

After UV irradiation, Polη forms nuclear foci that co-localize with PCNA (6,15,25–27,39,44). To determine the roles of each of Polη's three PIP boxes in foci formation, wild type and *pip* mutants bearing a FLAG-tag, all of which were used in the experiments shown in Figure 1, were transiently expressed in XP-V cells. After UV irradiation, localization of Polη and PCNA was visualized using anti-Polη and anti-PCNA antibodies, respectively. As shown in Figure 7A, Polη foci co-localized with PCNA were observed in all of the samples except for the *pip2* mutant. Because all of the proteins, including the *pip2* mutant, could be detected with similar efficiency by western blotting (Figure 1B) and immunostaining (Supplementary Figure S7), we conclude that PIP2, but not PIP1 or PIP3, plays a crucial role in foci formation along with PCNA.

Next, we examined the abilities of the Polη mutants to complement the UV sensitivity of XP-V cells (Figure 7B). Although the *pip* and *ubz* mutants of Polη have been analysed previously using such assays, the levels of complementation were inconsistent among studies (5–6,25–27,30). Complementation of the UV sensitivity of XP-V cells differs among clones stably expressing a particular mutant Polη, but is not correlated with the expression levels of Polη (26). To avoid such complexities due to differences among clones, we used for our survival assays a mixture of the cells that were transfected with pIRESneo2 carrying wild-type or mutant Polη and selected by G418. As shown in Figure 7B, the results indicated that the single and double *pip* mutants could complement the UV sensitivity of XP-V cells as efficiently as the wild type. By contrast, the *pip1 pip3 pip2* triple mutant exhibited clearly reduced complementation activity (Figure 7B).

In contrast to such subtle phenotypes of individual single and double *pip* mutants, the *ubz* single mutant exhibited a severe defect (Supplementary Figure S8) (6,26). However, because the *ubz* mutant accumulated poorly in the chromatin fraction (see Supplementary Figure S2) (26), we hypothesized that this defect could be attributed to secondary effects due to the *ubz* mutation. To investigate this possibility, we made use of a deletion mutant carrying the 1–511 region of Polη (PolηΔC) (2–3,32) (Figure 1A). Because PolηΔC lacks the nuclear localization signal (NLS) as well as PIP2 and UBZ, we introduced an artificial NLS at the C-terminus. Because of the lack of PIP2, PolηΔC was expected to have a lower ability to promote PCNA ubiquitination and fail to form foci in co-localization with PCNA. Those properties were confirmed *in vivo* and *in vitro* (Supplementary Figure S9). Nevertheless, PolηΔC retained the ability to accumulate in the chromatin fraction (Supplementary Figure S9D), in contrast to the *ubz* mutants of full-length Polη (Supplementary Figure S2), supporting the idea that some *ubz* mutations provoke secondary effects (5,30). More importantly, PolηΔC could complement the UV sensitivity of XP-V cells much better than the *ubz* mutant (Supplementary Figure S8). As shown in Figure 7B, the *pip1* and *pip3* derivatives of PolηΔC exhibited reduced complementation activity, and the *pip1 pip3* derivative of PolηΔC exhibited a severer defect than the *pip1 pip3 pip2* triple mutant of full-length Polη. The defects were not attributable to alterations in the sub-cellular localization (Supplementary Figure S9D). Together, these results suggest that PIP1, PIP3, PIP2 and UBZ exert additive and redundant effects that protect cells from the lethal effects of UV irradiation.

## DISCUSSION

Cellular functions of the respective motifs of Polη are routinely monitored in two ways: co-localization with PCNA and complementation of UV sensitivity of XP-V cells. In this study, we demonstrated that these two phenotypes are mediated by different PIP boxes and distinct modes of interaction with PCNA. We also showed that the diverse functions of PIP boxes are conserved in Polκ, but not in Polι.

### Functions of PIP boxes in ubiquitination of PCNA and foci formation

In this study, we found that Polη promotes mono-ubiquitination of PCNA in a manner dependent on PIP2 and to a lesser extent on PIP3, but independent of PIP1 *in vivo* (Figure 1B). These findings were perfectly correlated with the *in vitro* observations regarding promotion of PCNA mono-ubiquitination by purified proteins (Figure 2B, C). Together, the data strongly suggest that the intracellular accumulation of mUb-PCNA with ectopically expressed Polη is a consequence of direct promotion of *de novo* ubiquitination. Importantly, we found that DNA is an absolute requirement for Polη-dependent PCNA ubiquitination reactions *in vitro* (Figure 2A). We suggest that the mode of interaction between PIP3 or PIP2 and PCNA on DNA for the promotion of PCNA ubiquitination could act in such a way as an appropriate substrate for RAD6-(RAD18)_2_ catalysis, which was independent of the interaction between RAD18 and Polη *in vitro*. The partial involvement of the interaction in the promotion of PCNA ubiquitination *in vivo* (29) could be attributed to an additional function, such as recruitment of Polη to damage sites (15).

The contribution of individual Polη PIP boxes to co-localization with PCNA was correlated with the effect on promotion of PCNA ubiquitination. These effects could be largely attributed to PIP2 (Figures 1B, 2B, C and 7A). Polκ also promotes mono-ubiquitination of PCNA (Figures 4B and 5A, B) (29) and co-localizes with PCNA (42). Polι failed to promote mono-ubiquitination of PCNA (Figures 4E and 5C, D) and failed to co-localize with PCNA by itself (45,46). These results suggest that a large part of the function of the PIP2 box in foci formation is the promotion of PCNA mono-ubiquitination, which is a prerequisite for co-localization of polη with PCNA in nuclear foci. The following observation supports this idea: first, accumulation is dependent on RAD18 and its catalytic activity (15,29). Second, accumulation of Polη is barely detectable in a human cell line in which the PCNA^K164R^ mutant is expressed instead of endogenous PCNA (28), or in a PCNA^K164R^ knock-in murine cell line (23). Therefore, we suggest that one of the functions of PIP2 in foci formation is to promote mono-ubiquitination of PCNA. The resultant mUb-PCNA could stabilize Polη via interaction with UBZ, because mUb-PCNA has a higher affinity for Polη than unmodified PCNA (Supplementary Figure S6). Thus, even though UBZ is dispensable for the promotion of PCNA ubiquitination (at least *in vitro*), UBZ may still contribute to stable foci formation. This idea does not exclude another role for PIP2 in foci formation via direct interaction with PCNA. Indeed, among the three PIPs of Polη, only the PIP2 peptide has been shown to interact directly with PCNA in yeast two-hybrid and structural analyses (10,11). Furthermore, enhancement of the PIP2–PCNA interaction of Polκ by manipulations of its PIP2 sequence (10) improves foci formation (29,47). These results suggest that stable interactions with mUb-PCNA via both PIP and UBZ are required for detectable focus formation.

Interestingly, PIP3 and RIR1 share the same FF residues for binding to PCNA and REV1, respectively (40). Therefore, it is unlikely that PIP3 could act as a PIP box and RIR at the same time. However, Polη has two RIRs, RIR1 and RIR2 (40). So far, we have been unable to detect any defect in the polη-REV1 interaction in the *rir1* single mutant in cells (39) or in yeast-two hybrid assays (40) (Supplemental Figure S1). We believe that the PIP3-PCNA interaction or *pip3* mutation does not interfere with REV1-related function(s) because of the presence of RIR2.

### Functions of PIP and UBD in stimulating DNA synthesis

In this study, we demonstrated that all three PIP boxes of Polη play roles in stimulating DNA synthesis, although the mechanisms are different. We showed that PIP-less Polη has an intrinsic defect in accessing the PCNA-loaded 3′-end; the presence of PIP1 or PIP3, but not PIP2, compensates for this inhibition (Figure 3B). It is likely that both PIP1 and PIP3 stimulate DNA synthesis via direct interaction with PCNA, and that PIP2 facilitates the PIP1–PCNA and PIP3–PCNA interactions. Because PIP1 and PIP3 are proximal to the catalytic domain of Polη, the PCNA-Polη interaction via PIP1 or PIP3 may enable the active site of Polη to effectively bind the 3′-OH end of the primer-terminus, whereas the interaction via PIP2, located distant from the C-terminus, may be less efficient in this respect. This mechanism seems conserved in Polκ and Polι, since each PIP1 box located adjacent to the catalytic domain can stimulate DNA synthesis. More recently, during the preparation of this manuscript, another group reported that PIP1 in Polκ is responsible for PCNA stimulation of *in vitro* DNA synthesis (48).

We demonstrated for the first time that mUb-PCNA stimulates DNA synthesis by human Polη, ι and κ (Figure 6A–H). We showed that the UBZ-function of Polη is largely PIP1- and PIP3-dependent and slightly PIP2-dependent (Figure 6A–F), suggesting that adequate stimulation by mUb-PCNA requires interaction with at least one PIP box. Alternatively, an increased local concentration of Polη around the primer end, achieved through PIP–PCNA interactions, indirectly promotes the interaction between UBZ and the ubiquitin moiety. Collectively, these results suggest that interactions between mUb-PCNA and Polη stimulate DNA synthesis via diverse mechanisms.

### Functions of PIP and UBZ in Polη in enhancing survival after UV irradiation

All three PIP boxes and UBZ of Polη serve additive and redundant functions in enhancing survival after UV irradiation (Figure 7B). The survival rate *in vivo* (Figure 7B) correlated well with the level of stimulation of DNA synthesis by these mutants by PCNA or mUb-PCNA *in vitro* (Figure 6A–H). However, there were two exceptions. One was the *ubz* point mutant that exhibited severe defects (Supplementary Figure S8), which might be attributed to a secondary effect related to accumulation on chromatin (Supplementary Figure S2). Indeed, the deletion mutation of UBZ and PIP2 in PolηΔC restored the sub-cellular localization (Supplementary Figure S9D) and increased the ability to complement the UV sensitivity of XP-V cells relative to the *ubz* point mutant (Supplementary Figure S8). This partial contribution of UBZ is consistent with reports of PCNA ubiquitination-independent TLS in PCNA^K164R^ knock-in murine cells (22,23). The other exception was the *pip1 pip3* double mutant. The defect in complementation of UV sensitivity of XP-V cells by the *pip1 pip3* mutant was marginal (Figure 7B), although its DNA synthesis was poorly stimulated by mUb-PCNA *in vitro* (Figure 6E). Because the mutant is proficient in the promotion of PCNA ubiquitination and foci formation (Figures 1B, 2B and 7A), the elevated local concentration of the mutant could compensate for the defect in stimulation, and this could explain the minor defect of the *pip1 pip3* mutant *in vivo*. Overall, the data suggest that the level of stimulation of DNA synthesis by mUb-PCNA could directly affect the efficiency of TLS *in vivo*.

### A model for the cellular functions of PIP and UBD of Pol η/ι/κ in the orchestration of TLS

Taking the results of previous reports together with the *in vivo* and *in vitro* data obtained in this study, we propose a model for cellular functions of the various motifs of Polη/ι/κ (Figure 7C). We suggest that interactions between Polη/κ and PCNA constitute a network that regulates promotion of mono-ubiquitination and accumulation of Polη/ι/κ. PCNA is concentrated on DNA in close proximity to replication forks (21,35,49). In the model, Polη/κ are recruited to these locations via interactions with PIP boxes together with RAD18 (15,41,43). The initial PIP–PCNA interactions are too transient and unstable to be detected as foci, but the interaction turns PCNA into an appropriate substrate for RAD6-(RAD18)_2_ catalysis, which promotes ubiquitination of PCNA. The resultant mUb-PCNA recruits additional Polη/ι/κ molecules via interaction with both PIPs and UBDs. Because Polη has a much higher affinity for mUb-PCNA than Polκ and Polι (Supplementary Figure S6), it is likely that Polη is predominantly recruited. In the case of Polη, the interactions are sufficiently stable for detectable foci formation, but in the case of Polκ they are relatively weak. By contrast, Polι binds mUb-PCNA too weakly to form stable foci by itself. Increased local concentrations of Polη/κ further promote ubiquitination of PCNA. Consequently, Polη/κ and ubiquitinated PCNA robustly accumulate around the stalled primer ends until accumulation of Polη/κ is saturated. Any mutation that disrupts the regulatory network should attenuate the response *in vivo* via activities of de-ubiquitination enzymes. This model is compatible with the dynamic mobile properties of Polη/ι in cells (50).

Accumulation of Polη in replication foci plays a minor role in survival, as reflected by the observation that accumulation-defective PIP2 mutant could perfectly complement UV sensitivity of XP-V cells (Figure 7B) (27). In addition, PolηΔC with a C-terminal deletion encompassing UBZ and PIP2 also exhibited considerable complementation activity (Figure 7B). These results suggest that Polη can perform TLS of UV lesions without accumulating in foci. The minor contribution of foci formation to cellular function is also true in other members of the Y family. Although accumulation of Polι in replication foci is dependent on Polη (45,46), Polι appears to be functional in TLS of UV lesions in Polη-knockout mice (51). Deficiency in the Polη-dependent co-localization of REV1 with UV lesions does not affect survival, but does modulate mutagenesis (39). In this scenario, one of physiological functions of the accumulated mUb-PCNA could be to establish an order of recruitment for TLS polymerases, via dynamic interactions between mUb-PCNA and their PIPs and UBDs, depending on their respective affinities around the stalled primer ends (50). This possibility is compatible with the observation that Polκ can be induced by specific agents that produce DNA damage cognate for Polκ (52). Increasing the ratio of Polκ forces it to predominantly access specific DNA damage. We believe that this model provides an integrated picture of the cellular functions of various motifs of the Y-family DNA polymerases.

## Supplementary Material

SUPPLEMENTARY DATA
