# Supplementary material for: Different types of interaction between PCNA and PIP boxes contribute to distinct cellular functions of Y-family DNA polymerases
Source: Nucleic Acids Res. 2015 Jul 13;43(16):7898–910. doi: 10.1093/nar/gkv712 (PMC4652755; doi:10.1093/nar/gkv712)
Supplement: SUPPLEMENTARY DATA [file supp_43_16_7898__index.html]

Different types of interaction between PCNA and PIP boxes contribute to distinct cellular functions of Y-family DNA polymerases — Different types of interaction between PCNA and PIP boxes contribute to distinct cellular functions of Y-family DNA polymerases — SUPPLEMENTARY DATA 

# Different types of interaction between PCNA and PIP boxes contribute to distinct cellular functions of Y-family DNA polymerases

## SUPPLEMENTARY DATA

- SUPPLEMENTARY DATA
