# Supplementary material for: Different types of interaction between PCNA and PIP boxes contribute to distinct cellular functions of Y-family DNA polymerases
Source: Nucleic Acids Res. 2015 Jul 13;43(16):7898–910. doi: 10.1093/nar/gkv712 (PMC4652755; doi:10.1093/nar/gkv712)
Supplement: SUPPLEMENTARY DATA [file supp_gkv712_nar-01199-d-2015-File009.docx]

**Supplementary Figure S1.** Yeast two-hybrid analysis of interactions between Polη and PCNA. *POLH* fragments with or without the indicated PIP mutations (see Figure 1A) were used. The REV1-CTD (C-terminal domain), which interacts with REV1-interacting regions (RIRs) (40), was used in parallel as a control. Assays were performed as described previously (40).

**Supplementary Figure S2.** Western blot analysis of cells expressing *ubz* mutants of Polη. XP-V cells were transfected with plasmids expressing FLAG-Polη (wt) or the indicated *ubz* mutants and incubated for 24 hours. The indicated fractions were separated as described in Materials and Methods and subjected to western blotting with anti-PCNA and anti-Polη antibodies. WCL, whole-cell lysates.

**Supplementary Figure S3.** Purified recombinant Polη (A), histidine-tagged Polκ (B), Polι (C), and mUb-PCNA (D). Five hundred nanograms of each indicated protein were loaded onto an SDS 5–20% gradient polyacrylamide gel and the gel was stained with CBB.


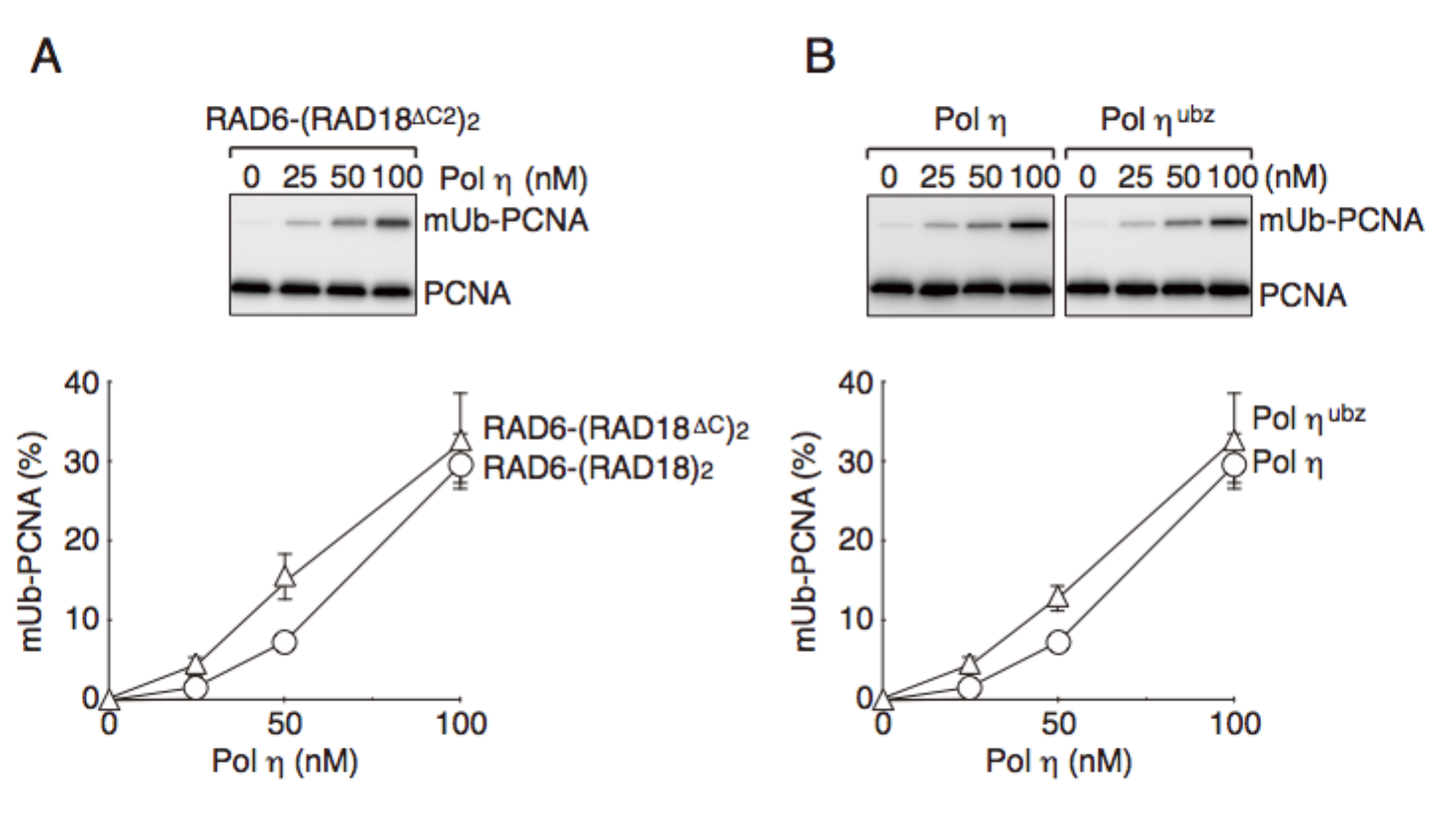


**Supplementary Figure S4.** Promotion of mono-ubiquitination of PCNA by Polη *in vitro*. **(A)** Promotion of mono-ubiquitination of PCNA by Polη with a mutant RAD6-(RAD18)_2_ complex, RAD6-(RAD18^∆C2^)_2_, which is truncated to remove the Polη-interacting domain (15,34). **(B)** Promotion of mono-ubiquitination of PCNA by Polη^ubz^. The ubiquitination assays were performed as shown in Figure 2. Relative amounts of ubiquitinated PCNA were measured from the gel images of three independent experiments, and average values are plotted in the graph. Error bars show SD. The titration curves for wild type RAD6-(RAD18)_2_ with wild type Polη shown as a control are the same as those in Figure 2B.


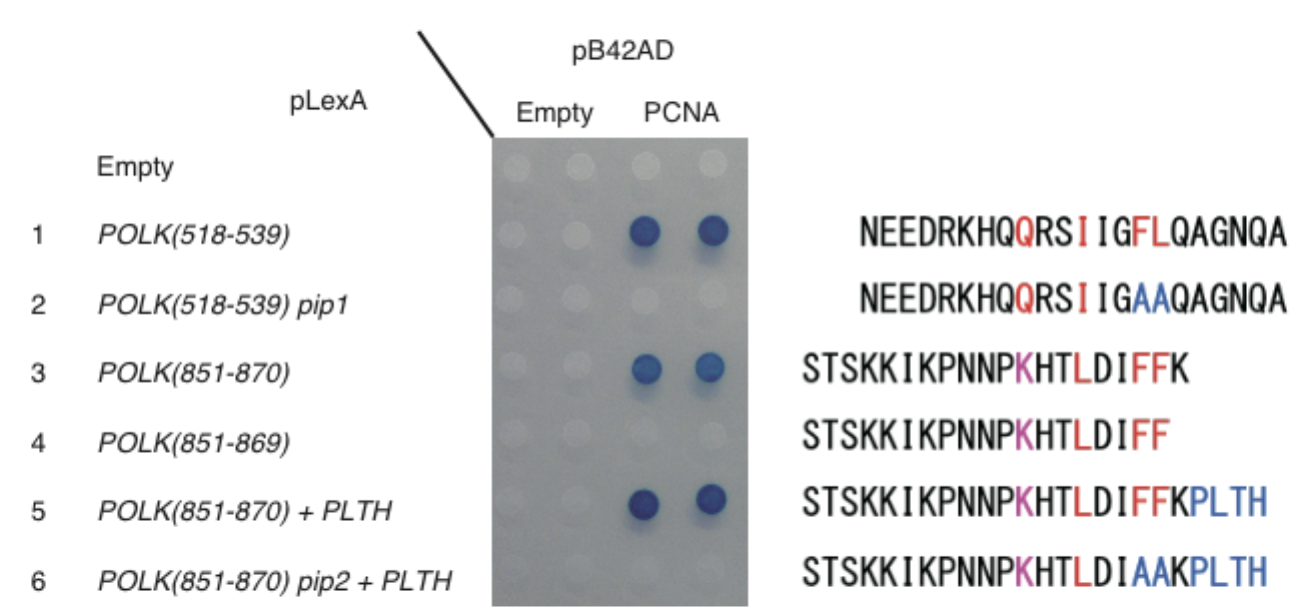


**Supplementary Figure S5.** Yeast two-hybrid analysis of interactions between PIP boxes of Polκ and PCNA. PIP fragments with or without the indicated amino acid changes were used (40). 1, a wild-type PIP1 fragment (518-539); 2, fragment 1 with the *pip1* mutation (Figure 4A); 3, a wild-type PIP2 fragment (851–870); 4, another PIP2 fragment missing the extreme C-terminal Lys residue; 5, fragment 3 with a PLTH sequence added to make it similar to the C-terminal sequence of Polη containing PIP2; and 6, fragment 5 with the *pip2* mutation (Figure 4A). The amino acid sequences are shown to the right. Note that, in the previous experiments using the SPR (Surface Plasmon Resonance) method (10), the interaction between PCNA and the PIP2 fragment of Polκ was detected only when the PLTH sequence was present in the C-terminus of Polκ. The present data indicate that the yeast two-hybrid assay is more sensitive than SPR and that the last Lys residue on the C-terminal side of the PIP-box is also essential for the PCNA-interaction (42).

**Supplementary Figure S6.** Physical interactions between mono-ubiquitinated PCNA and polymerases. The indicated his-tagged polymerases were immobilized on magnetic beads and incubated with PCNA (lanes 2–4) or mUb-PCNA (lanes 7–9). Lanes 5 and 10 are buffer controls. In lanes 1 and 6, PCNA and mUb-PCNA, respectively, were loaded as standards at 4% of the amounts used in the assays.

**Supplementary Figure S7.** A control experiment for Figure 7A. Cells shown in Figure 7A were fixed without Triton-X 100 treatment or UV irradiation, and FLAG-Polη and PCNA were visualized by immunostaining with anti-Polη and anti-PCNA antibodies, respectively. Nuclei were stained with Hoechst 33342. Scale bars represent 50 μm.

**Supplementary Figure S8.** UV sensitivity of XP-V cells stably expressing the *ubz* mutant FLAG-Polη^ubz^. Cells were irradiated with the indicated dose of UVC, incubated with 1 mM caffeine for 4 days, and their viabilities were measured. Error bars show SD from three independent experiments. The survival curves of Polη, PolηΔC, and the vector, shown as controls, are the same as those in Figure 7B.


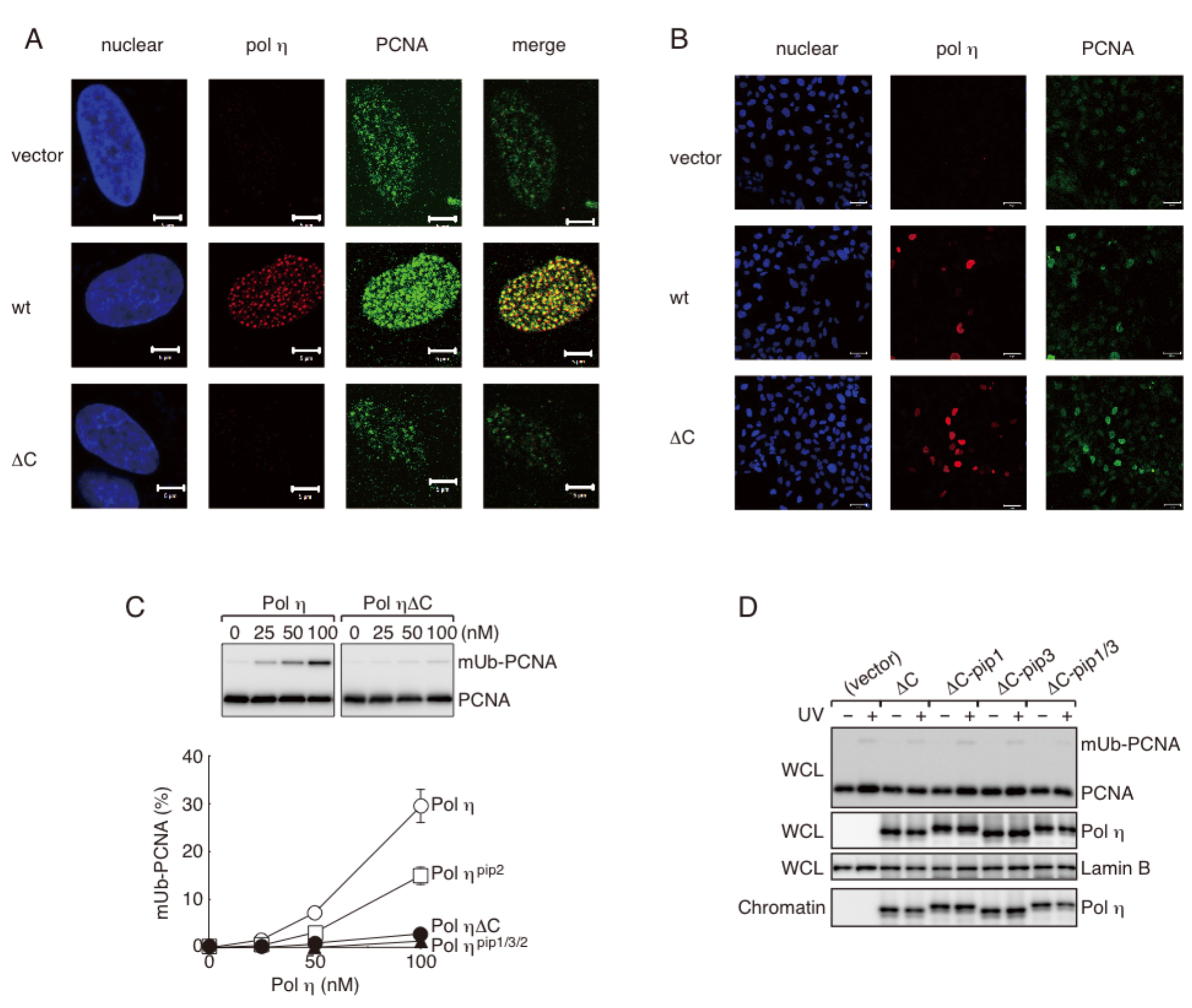


**Supplementary Figure S9.** Analysis of PolηΔC. **(A)** Defect in the co-localization of PolηΔC with PCNA. XP-V cells were transiently transfected with each plasmid to express FLAG-Polη or FLAG-PolηΔC. After UV irradiation, FLAG-Polη and PCNA were visualized by immunostaining with anti-FLAG and anti-PCNA antibodies, respectively. Nuclei were stained with Hoechst 33342. Scale bars represent 5 μm. **(B)** Control experiment for (A). Cells shown in (A) were fixed without Triton-X 100 treatment or UV irradiation. FLAG-Polη and PCNA were visualized by immunostaining with anti-FLAG and anti PCNA antibodies, respectively. Nuclei were stained with Hoechst 33342. Scale bars represent 50 μm. **(C)** Defect in the promotion of mono-ubiquitination of PCNA by PolηΔC. The ubiquitination assays were performed as shown in Figure 2. Relative amounts of ubiquitinated PCNA were measured from gel images of three independent experiments, and the average values are plotted in the graph. Error bars show SD. The titration curves of Polη, Polη^pip2^, and Polη^pip1/3/2^, shown as controls, are the same as those in Figure 2B. (D) Western blot analysis of PolηΔC-expressing cells. XP-V cells were transfected with plasmids to express FLAG-PolηΔC or the indicated *pip* mutants of PolηΔC and incubated for 24 hours. Indicated fractions were separated as described in Materials and Methods and subjected to western blotting with anti-PCNA and anti-Polη antibodies. WCL, whole-cell lysates.
